# An outcome prediction model for exsanguinating patients with blunt abdominal trauma after damage control laparotomy: a retrospective study

**DOI:** 10.1186/1471-2482-14-24

**Published:** 2014-04-28

**Authors:** Shang-Yu Wang, Chien-Hung Liao, Chih-Yuan Fu, Shih-Ching Kang, Chun-Hsiang Ouyang, I-Ming Kuo, Jr-Rung Lin, Yu-Pao Hsu, Chun-Nan Yeh, Shao-Wei Chen

**Affiliations:** 1 Department of Trauma and Emergency Surgery, Chang Gung Memorial Hospital, Chang Gung University, 5, Fu-Hsing Street, Kwei Shan Township, Taoyuan, Taiwan; 2 Graduate Institute of Clinical Medical Sciences, Chang Gung University, Taoyuan, Taiwan; 3 Clinical Informatics and Medical Statistics Research Center, Chang Gung University, Taoyuan, Taiwan; 4 Department of General Surgery, Chang Gung Memorial Hospital, Taoyuan, Taiwan; 5 Department of Cardiothoracic and Vascular Surgery, Chang Gung Memorial Hospital, Taoyuan, Taiwan

**Keywords:** Blunt abdominal trauma, Damage control laparotomy, Damage control surgery

## Abstract

**Background:**

We present a series of patients with blunt abdominal trauma who underwent damage control laparotomy (DCL) and introduce a nomogram that we created to predict survival among these patients.

**Methods:**

This was a retrospective study. From January 2002 to June 2012, 91 patients underwent DCL for hemorrhagic shock. We excluded patients with the following characteristics: a penetrating abdominal injury, age younger than 18 or older than 65 years, a severe or life-threatening brain injury (Abbreviated Injury Scale [AIS] ≥ 4), emergency department (ED) arrival more than 6 hours after injury, pregnancy, end-stage renal disease, or cirrhosis. In addition, we excluded patients who underwent DCL after ICU admission or later in the course of hospitalization.

**Results:**

The overall mortality rate was 61.5%: 35 patients survived and 56 died. We identified independent survival predictors, which included a preoperative Glasgow Coma Scale (GCS) score < 8 and a base excess (BE) value < -13.9 mEq/L. We created a nomogram for outcome prediction that included four variables: preoperative GCS, initial BE, preoperative diastolic pressure, and preoperative cardiopulmonary cerebral resuscitation (CPCR).

**Conclusions:**

DCL is a life-saving procedure performed in critical patients, and devastating clinical outcomes can be expected under such dire circumstances as blunt abdominal trauma with exsanguination. The nomogram presented here may provide ED physicians and trauma surgeons with a tool for early stratification and risk evaluation in critical, exsanguinating patients.

## Background

Damage control laparotomy (DCL) has been adopted as a standard treatment for patients with life-threatening injuries following major trauma. DCL is an initial laparotomy performed to address hemorrhage and contamination, and it may include gauze packing for hemorrhage control, vascular pedicle ligation, contamination control, and the establishment and maintenance of an abdominal wall opening covered with plastic, with or without a vacuum device [1,2]. Following this initial emergent management, the patient is admitted to the intensive care unit (ICU) to correct hypothermia, coagulopathy, acidosis, and electrolyte imbalances. Within 48 to 72 hours after the first laparotomy, a second laparotomy is performed for definitive treatment and abdominal closure. DCL was first applied in patients with hepatic injuries during the early 20^th^ century, and this technique was further developed during the 1980s [2,3]. Currently, DCL is widely used in the emergency setting for patients with uncontrolled intra-abdominal bleeding or severely contaminated intestinal or urological trauma.

Blunt abdominal trauma is common in Taiwan. It accounted for 96% of all hospitalized abdominal trauma patients in our institution in 2011. Previous studies have looked at either penetrating injuries [1,4] or abdominal trauma as a whole [5-7], but as of yet no studies have focused on blunt abdominal trauma. We present a series of patients with blunt abdominal trauma, all of whom underwent DCL with packing to control massive intra-abdominal bleeding. In addition, we introduce a nomogram that we developed to help predict outcomes among these patients.

## Methods

### Clinical setting

Chang Gung Memorial Hospital (CGMH) is a level I trauma center in northern Taiwan. From May 2008 to June 2012, 1203 patients who had sustained abdominal trauma and 336 patients who underwent surgery (either laparotomy or a laparoscopic procedure) were treated at CGMH. A standard protocol for treating blunt abdominal trauma has been in place at CGMH for over 10 years. During that time, emergent transarterial embolization (TAE) has been widely applied in our institution and has been made available on a 24-hour basis. At CGMH, over 70% of patients who need TAE are sent to the angiographic room within 1 hour in accordance with patients’ critical levels. Approximately 90% of patients with solid organ injuries (including hepatic, renal, and splenic) are managed nonoperatively, with a failure rate of less than 2%. Among patients with intra-abdominal bleeding, laparotomies are performed only in cases of refractory hemorrhagic shock, a transient response to resuscitation, multiple bleeding sites with a difficult TAE approach, and either TAE failure or a transient effect of TAE.

### Exclusion criteria

In this study, we excluded patients with the following characteristics: a penetrating abdominal injury, age younger than 18 or older than 65 years, a severe or life-threatening brain injury (Abbreviated Injury Scale [AIS] ≥ 4), emergency department (ED) arrival more than 6 hours after injury, pregnancy, end-stage renal disease, or cirrhosis. We also excluded patients with a concurrent chest injury and indications for thoracotomy or a pelvic injury with indications for pre-peritoneal packing. In addition, we excluded patients who underwent DCL after ICU admission or later during the course of hospitalization. Only patients who had sustained a blunt abdominal trauma and were transported to the operating room (OR) directly from the ED were enrolled.

### Study design

This retrospective study was approved by the local institutional review board (IRB) of CGMH. Forty-five patients fulfilled the study criteria from May 2008, when the CGMH Trauma Registration System began, to May 2012. These patients all sustained abdominal trauma and underwent DCL with gauze packing. For the pre-registration period, January 2002 to April 2008, we accessed the operating room information system to retrieve the list of patients who underwent emergent laparotomy and fulfilled our study criteria. The medical and surgical data for the 46 eligible patients were then extracted. In total, 91 patients were enrolled for further statistical analysis.

The patients’ surgical records and radiologic reports were evaluated by two surgical residents and two attending surgeons who assessed the accuracy of the extracted information. The extracted and analyzed data included each patient’s demographic data, the mechanism of trauma, initial status in the ED, initial laboratory data, the quantity of blood transfused, status upon discharge from the ED, injury severity score (ISS), revised trauma score (RTS), surgical condition, diagnosis, and outcome. All of the patients were categorized into two groups: the survival group (n = 35) and the mortality group (n = 56). These groups were compared using univariate analysis, and selected resulting factors of significance were further analyzed with multivariate analysis and then used in the nomogram creation.

### Statistical analysis

We used R (version 2.15.1) open source statistical software with the appropriate packages for statistical analysis. The Student’s *t*-test was used to evaluate numerical variables, and the *χ*^2^ test was used for nominal data. Levene’s test was used to correct for intergroup variation before the application of the Student’s *t*-test. A receiver operating characteristic (ROC) curve analysis was performed for continuous factors of significance prior to applying logistic regression with forward selection.

The creation of the nomogram was based on an established model. In our study, the nomogram was created based on the results of logistic regression with forward stepwise selection. Each factor in the logistic regression model will be later used for nomogram creation. The factor with the highest odds ratio is given a score of 100 points. Other factors receive their own scores according to the value of their individual odds ratios relative to the highest odds ratio.

Because of the relatively small number of cases in our study, we conducted only internal validation using the bootstrapping method. A survival analysis was conducted using the Kaplan-Meier method.

## Results

**Figure 1:**
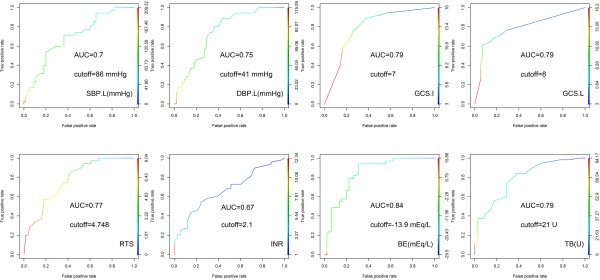
**ROC curve analysis.** SBP.L: systolic blood pressure when leaving ED; DBP.L: diastolic blood pressure when leaving ED; GCS.I: initial Glasgow Coma Scale; GCS.L: Glasgow Coma Scale when leaving ED; RTS: revised trauma score; INR: international normalized ratio; BE: base excess; TB: transfusion of packed red blood cell and whole blood at both ED and OR.

The demographic data and ED arrival status of the patients in the two groups are compared and summarized in Table 1. Comparisons between the groups in terms of patient status upon ED discharge and other surgical conditions are summarized in Tables 2 and 3. Thirteen factors significantly differed between the groups: RTS, respiratory rate before OR transportation, systolic blood pressure (SBP) and diastolic blood pressure (DBP) before OR transportation, initial Glasgow Coma Scale (GCS), GCS before OR transportation, initial laboratory findings (INR, arterial pH, HCO_3_^-^ level, and base excess), the volume of whole blood transfused in the OR, the volume of total blood (packed red blood cells and whole blood) transfused in both the ED and OR, and the performance of cardiopulmonary cerebral resuscitation (CPCR) in the ED (in our series, the duration of CPCR was less than 15 minutes in all cases). To enable practical application of the nomogram, we transformed these continuous factors into categorical data according to the ROC curve analysis illustrated in Figure 1.

**Table 1:** Demographic data and initial status of patients

	**Survival (n = 35)**	**Mortal (n = 56)**	***P *** **value**
Age	33.7 ± 16.62	39.6 ± 15.87	0.092
Gender (M/F)	26/9	45/11	0.496
Transferred (Y/N)	28/7	36/20	0.110
Season			
Spring	5	17	0.264
Summer	9	13	
Fall	11	17	
Winter	10	9	
Accident to ED (mins)	184.8 ± 127.93	139.7 ± 92.35	0.073
Initial body temperature (°C)	35.9 ± 1.32	34.3 ± 4.86	0.075
Initial RR (/min)	21.8 ± 6.95	19.6 ± 11.37	0.251
Initial HR (/min)	111.1 ± 27.72	107.8 ± 42.27	0.653
Initial SBP (mmHg)	84.3 ± 40.50	69.7 ± 53.46	0.169
Initial DBP (mmHg)	54.7 ± 28.93	41.7 ± 38.79	0.090
Initial GCS	11.8 ± 3.94	6.6 ± 4.47	0.000
RTS	6.08 ± 1.40	3.95 ± 2.30	0.000

Transferred: patient was sent to local hospital initially; *ED* emergency department; *RR* respiratory rate; *HR* heart rate; *SBP* systolic blood pressure; *DBP* diastolic blood pressure; *GCS* Glasgow Coma Scale; *RTS* Revised Trauma Score. All continuous data presented with standard deviation.

**Table 2:** Initial laboratory data, patient status before leaving ED, and transfusion amount at ED

	**Survival (n = 35)**	**Mortal (n = 56)**	***P *** **value**
Hb (g/dL)	9.7 ± 2.60	9.3 ± 3.30	0.584
Hct (%)	28.4 ± 7.74	28.6 ± 9.80	0.688
INR	1.82 ± 0.68	3.07 ± 2.89	0.005
pH	7.26 ± 0.11	7.07 ± 1.97	0.000
BE (mEq/L)	-8.28 ± 4.67	-15.34 ± 7.33	0.000
HCO_3_ (mEq/L)	18.6 ± 4.04	14.6 ± 5.26	0.000
CPCR at ED	0	21	0.000
RR before leaving ED (/min)	21.9 ± 4.60	18.8 ± 6.71	0.061
HR before leaving ED (/min)	120.0 ± 30.02	107.1 ± 42.50	0.126
SBP before leaving ED (mmHg)	102.1 ± 35.28	77.1 ± 36.67	0.002
DBP before leaving ED (mmHg)	58.9 ± 20.73	40.0 ± 27.75	0.000
GCS before leaving ED	9.3 ± 4.85	4.3 ± 2.99	0.000
PRBC at ED (U)	5.2 ± 5.85	5.2 ± 6.99	0.982
FFP at ED (U)	1.7 ± 2.38	2.1 ± 4.60	0.642
PLT at ED (U)	0.0 ± 0.00	1..0 ± 4.03	0.069
WB at ED (U)	1.7 ± 2.63	2.7 ± 3.67	0.157
Time to OR (/min)	128.7 ± 118.05	93.3 ± 99.19	0.128
Perioperative TAE	9	13	0.786

*Hb* hemoglobin; *Hct* hematocrit; *INR* international normalized ratio; *BE* base excess; *CPCR* cardiopulmonary cerebral resuscitation; *ED* emergency department; *RR* respiratory rate; *HR* heart rate; *SBP* systolic blood pressure; *DBP* diastolic blood pressure; *GCS* Glasgow Coma Scale; *PRBC* packed red blood cells; *FFP* Fresh frozen plasma; *PLT* platelet; *WB* whole blood; *OR* operation room; *TAE* transarterial embolization. All continuous data presented with standard deviation.

**Table 3:** Surgical finding and OR transfusion

	**Survival (35)**	**Mortal (56)**	***P *** **value**
Major bleeder			
Liver	14	33	0.272
Spleen	3	4	
Mesentery	5	3	
Kidney	2	0	
Retroperitoneum	1	0	
Multiple	8	12	
Others	2	4	
PRBC at OR	10.4 ± 12.36	15.0 ± 17.64	0.151
FFP at OR	9.2 ± 7.00	9.8 ± 9.09	0.749
PLT at OR	8.8 ± 11.74	9.7 ± 9.09	0.748
WB at OR	3.8 ± 5.42	13.9 ± 12.52	0.000
Total PRBC + WB*	21.0 ± 11.78	36.0 ± 19.58	0.000
ISS	33.1 ± 15.06	33.7 ± 15.09	0.874

*PRBC* packed red blood cells; *FFP* Fresh frozen plasma; *PLT* platelet; *WB*: whole blood; *OR* operation room; *ISS* Injury Severity Score. All continuous data presented with standard deviation. * total whole blood and PRBC transfused at ED and OR.

**Figure 2:**
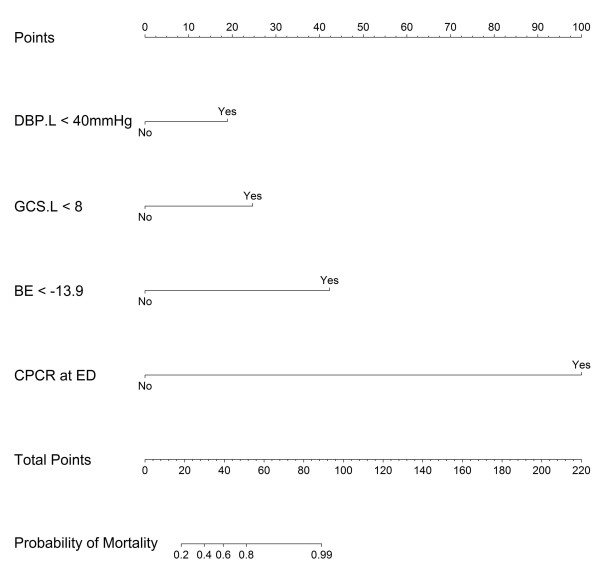
**Nomogram for outcome prediction.** DBP.L: diastolic blood pressure when leaving ED; GCS.L: Glasgow Coma Scale when leaving ED; BE: base excess; CPCR: cardiopulmonary cerebral resuscitation.

**Figure 3:**
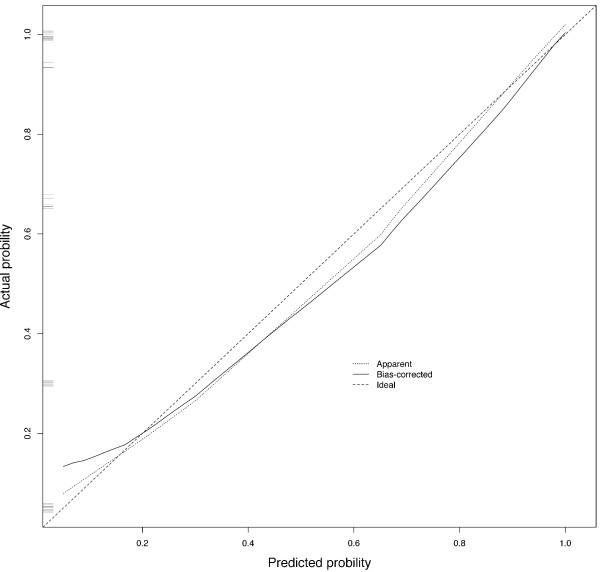
Calibration curve for nomogram.

Logistic regression with forward selection was used to analyze nine of the significant factors under univariable analysis (RTS, SBP and DBP before OR transportation, initial GCS, GCS before OR transportation, INR, base excess, CPCR at ED, and total blood transfused in both the ED and OR). There are 4 factors retained in the final equation (GCS less than 8 when leaving ED, BE less than 13.9 mEq/L, DBP less than 40 mmHg when leaving ED, and CPCR at ED) and 2 of them are noted with statistical significance while the other 2 are retained in equation for optimal *R*^2^ achievement. All the 4 factors in the logistic regression model are necessary for nomogram creation. A summary of the logistic regression, including the odds ratios, is shown in Table 4. We applied the model produced by logistic regression to develop a nomogram for outcome prediction (Figure 2) along with its calibration curve (Figure 3). The C-index for the internal validation was 0.946.

**Table 4:** Result of multivariable logistic regression

	**Odds ratio**	**P values**
GCS < 8	7.77	0.020
BE < -13.9	28.50	0.000

*GCS* Glasgow Coma Scale; *BE* base excess. 95% CI: 95% confidence interval for odds ratio.

**Figure 4:**
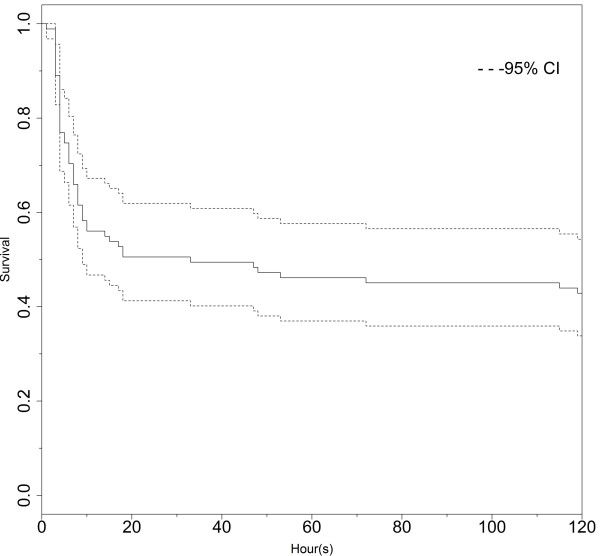
Survival analysis.

A survival analysis was also performed; the Kaplan-Meier survival curve is shown in Figure 4. The mortality rate in this series was 61.5% (n = 56). Among the patient deaths, 50% occurred within 8 hours of arrival in the ED, and 80% occurred within 24 hours of arrival in the ED.

## Discussion

Damage control laparotomy is a life-saving procedure indicated for patients who typically do not have any other treatment options. In addition to critical trauma, DCL can be used to treat severe intra-abdominal sepsis or uncontrolled and unexpected intra-abdominal bleeding. The aim of DCL is to control hemorrhage and contamination during the early, life-threatening period of such an emergency. After DCL, the patient’s condition can be stabilized before he or she undergoes the next step of definitive surgical treatment. Even with the development of new strategies to manage and resuscitate patients with severe trauma [8-10], DCL continues to play an important role in trauma care. In this study, we focused solely on patients who had sustained blunt abdominal trauma with exsanguination. All of the patients in our series underwent surgery with packing to achieve temporary hemostasis.

The overall mortality rate in our study was 61.5%, which is similar to previously reported results [5,11]. Although 61.5% may appear high compared with the mortality rate of approximately 20% recently reported by Cotton et al. [12], this discrepancy may be related to the different clinical conditions of the two studies. We included only blunt abdominal trauma with hemorrhagic shock, whereas Cotton et al. included all patients who underwent DCL. Neither the type of injury (blunt or penetrating; only 4% of the abdominal traumas in our institution were penetrating) nor the indication for DCL (bleeding control or contamination control) was specifically mentioned in Cotton’s report. In addition, patients who received CPCR for over 5 minutes and patients who died in the OR were excluded from the Cotton study. Although there have been other reports regarding the outcomes of DCL, none have been similar to our study in terms of the patient population examined. Therefore, the differing clinical settings of these studies prevent objective comparisons.

Because rapid, dynamic changes in the clinical courses of severely injured patients are frequently observed, the results of studies regarding their clinical outcomes are often heterogeneous. In addition, the classical trimodal distribution of trauma deaths implies that marked changes in the probability of death occur within several hours of injury. We used strict criteria in the selection of our study subjects to prevent the interference of any confounding factors. These criteria were chosen not only to limit the subjects to patients treated in the ED (because other hospital departments may follow different clinical routines) but also to exclude subjects who arrived in our ED more than 6 hours after injury.

The purpose of our study was to establish a prediction system that would help ED physicians and surgeons to formulate more precise impressions regarding expected clinical outcomes in the initial stages of patient care. We established our model using logistic regression with forward selection to retain the most significant factors. This approach also proved practical for the later creation of the nomogram; if logistic regression without this stepwise approach had been used to create the nomogram, the inclusion of an excessive number of variables would have made the nomogram impractical. In addition, we excluded from the logistic regression several factors that were deemed significant in univariate analysis. Respiratory rate was excluded because of its narrow physiological range and poor linear correlation with physical status. Although the BE, HCO_3_^-^ level, and pH value are all indicators of acidosis, we chose to analyze only BE because of previous reports of its superior predictive power [13-15]. Ultimately, four factors were retained in the equation.

Several studies have reported predictive factors for outcomes after DCL. One study proposed that hyperthermia and the arterial pH level at the time of ICU admission, as well as the volume of blood transfused within 24 hours, are the best predictors of post-DCL outcomes [5]. However, the results of these studies differ, due in large part to variations in their clinical settings. In addition, some studies have separately examined poor prognostic factors, such as cirrhosis of the liver and advanced age [16,17]. In our study, we employed strict criteria to exclude subjects with significant medical disease. The independent factors affecting the outcomes of patients in our study included BE and GCS before OR arrival. Both of these factors reflect the presence of shock and hypoperfusion. The most unique aspect of our study is that all of the analyzed factors can be assessed upon completion of DCL, which enables our model to be applied during the early stages of hospitalization.

The ability to assess all of these factors during the early stages of a patient’s clinical course was advantageous in producing an early prediction tool for DCL patients with hemorrhagic shock. We simplified the significant factors by using univariate analysis of binary factors. Meanwhile, the use of stepwise logistic regression enabled us to eliminate less significant factors from the multivariate analysis. The goal of this approach was the construction of a simple, handy nomogram for predicting outcomes at an early stage. In addition to BE and the GCS before OR transportation, our model includes DBP before OR transportation and CPCR as factors. DBP is an important factor related to coronary perfusion, as the diastolic time determines the coronary perfusion time [18]. The decrease in DBP caused by hemorrhagic shock can cause a devastating impairment in cardiac function. Finally, the need for CPCR reflects a combination of all of the risks present and can reasonably be considered an indicator of a poor prognosis.

There were some unavoidable limitations to our study. First, our patient care strategies evolved over the 10-year study period, and advancements in treatment concepts and strategies may have affected patient outcomes [9,12,19]; these changes placed the patients treated 10 years ago and those treated more recently in different contexts. The most obvious change occurred in our transfusion strategy. Currently, component therapy transfusion is the mainstream protocol for trauma patients with hemorrhagic shock, as mentioned in the Advanced Trauma Life Support (ATLS) protocol. However, this concept was not popular a decade ago; for example, in data from the pre-registration period of our study versus that of the CGMH trauma registration system, 13.6 U vs. 6.6 U of whole blood were used, respectively, in the OR per patient. Second, the number of subjects enrolled in our study was limited, which was an obstacle to the creation of our model and nomogram. Although internal validation revealed an acceptable fit of the model, future external validation is needed to further evaluate the efficacy of our nomogram. Overall, although our study had some drawbacks in terms of sample collection and the small sample size (which created a statistical disadvantage), it is the first to introduce the nomogram prediction model in this genre of surgery.

## Conclusion

In conclusion, although a high death toll remains, DCL is a potentially life-saving procedure with the potential to mitigate the devastating clinical outcomes that can be expected under the dire circumstances of blunt abdominal trauma with exsanguination. The nomogram that we have proposed here may provide ED physicians and trauma surgeons with a tool for early stratification and risk evaluation in these critical, exsanguinating patients.

## Competing interests

No personal competing interests of our co-authors or any funding from either public sector or private sector is related to the study.

## Authors’ contributions

SW provided the idea of study design and manuscript writing, CL summarized the clinical data, formed the table, and helped structure the manuscript. CF provided the effort for critical revision. SK provided the concept of study formation and provided opinion for critical revision. CO checked and reviewed the collected data from database. IK checked and reviewed the collected data from database. JL provided the consultation of statistical analysis. HP helped the study design and critical revision. CY helped the revision and statistical analysis. SC helped in data collection from reviewed the data of early trauma data. All authors read and approved the final manuscript.

## Pre-publication history

The pre-publication history for this paper can be accessed here:

http://www.biomedcentral.com/1471-2482/14/24/prepub
